# How genetic data improve the interpretation of results of faecal glucocorticoid metabolite measurements in a free-living population

**DOI:** 10.1371/journal.pone.0183718

**Published:** 2017-08-23

**Authors:** Maik Rehnus, Rupert Palme

**Affiliations:** 1 Swiss Federal Research Institute for Forest, Snow and Landscape Research WSL, Zürcherstrasse 111, Birmensdorf, Switzerland; 2 WILDTIER SCHWEIZ, Winterthurerstrasse 92, Zurich, Switzerland; 3 Department of Biomedical Sciences, University of Veterinary Medicine, Veterinärplatz 1, Vienna, Austria; Centre for Cellular and Molecular Biology, INDIA

## Abstract

Measurement of glucocorticoid metabolites (GCM) in faeces has become a widely used and effective tool for evaluating the amount of stress experienced by animals. However, the potential sampling bias resulting from an oversampling of individuals when collecting “anonymous” (unknown sex or individual) faeces has rarely been investigated. We used non-invasive genetic sampling (NIGS) to investigate potential interpretation errors of GCM measurements in a free-living population of mountain hares during the mating and post-reproductive periods. Genetic data improved the interpretation of results of faecal GCM measurements. In general GCM concentrations were influenced by season. However, genetic information revealed that it was sex-dependent. Within the mating period, females had higher GCM levels than males, but individual differences were more expressed in males. In the post-reproductive period, GCM concentrations were neither influenced by sex nor individual. We also identified potential pitfalls in the interpretation of anonymous faecal samples by individual differences in GCM concentrations and resampling rates. Our study showed that sex- and individual-dependent GCM levels led to a misinterpretation of GCM values when collecting “anonymous” faeces. To accurately evaluate the amount of stress experienced by free-living animals using faecal GCM measurements, we recommend documenting individuals and their sex of the sampled population. In stress-sensitive and elusive species, such documentation can be achieved by using NIGS and for diurnal animals with sexual and individual variation in appearance or marked individuals, it can be provided by a detailed field protocol.

## Introduction

Measurement of glucocorticoid metabolites (GCM) has become a widely used and effective tool for evaluating the amount of stress experienced by animals [1–4]. The advantage of faecal GCM measurements is that samples can be collected easily without any need to handle the animal. Thus, the sampling process is almost feedback free and is therefore appropriate for evaluating stress faced by free-living wild animals [5]. However, besides various factors, GCM excretion may also depend on the sex of sampled individuals and on the season [6–10] and thus it is important to know the composition of the sample population to correctly interpret GCM measurements.

The extraction of genetic material from faeces, hair and other sources of DNA enables the collection of genotype data and sex ratio in wildlife populations without the need to handle, capture or even observe individual animals [11]. Such non-invasive genetic sampling (NIGS) has become a popular method for wildlife biologists and managers [12–14], especially of elusive and stress-sensitive species [15, 16]. The application of NIGS in combination with GCM measurements has the potential to allow for an evaluation of stress faced by free-living wild animals. However, to our knowledge, the combination of NIGS and GCM has never been tested on an individual level in free-living animals, but was only used for species or sex identification [17–20].

Our model species, the mountain hare (*Lepus timidus*), is a perfect species for testing the suitability of the combination of GCM and NIGS methods, because both GCM and NIGS have recently been developed specifically for it [16, 21–23]. The mountain hare is an elusive species that is nocturnally active, has no sexual dimorphism and is sensitive to disturbance [24, 25]. It is a non-territorial species and individual home ranges show considerable overlap [26]. This increases the risk that individual mountain hares may be oversampled when “anonymous” faeces are collected.

In this study, we addressed how knowledge (derived from NIGS) about the composition of the sampled population influences the interpretation of results of GCM measurements taken during the mating and post-reproductive periods. First, we present the results of GCM values derived from standard sample collections (“anonymous” samples without considering the sex or the individual). Next, we compare GCM values while considering the sex. We then evaluate our results of GCM measurements per season and sex based on the knowledge of genotyped individuals. Finally, we discuss the use of the combination of NIGS and GCM to improve the interpretation of results of faecal glucocorticoid metabolite measurements in a free-living population.

## Material and methods

### Study area

**Fig 1:**
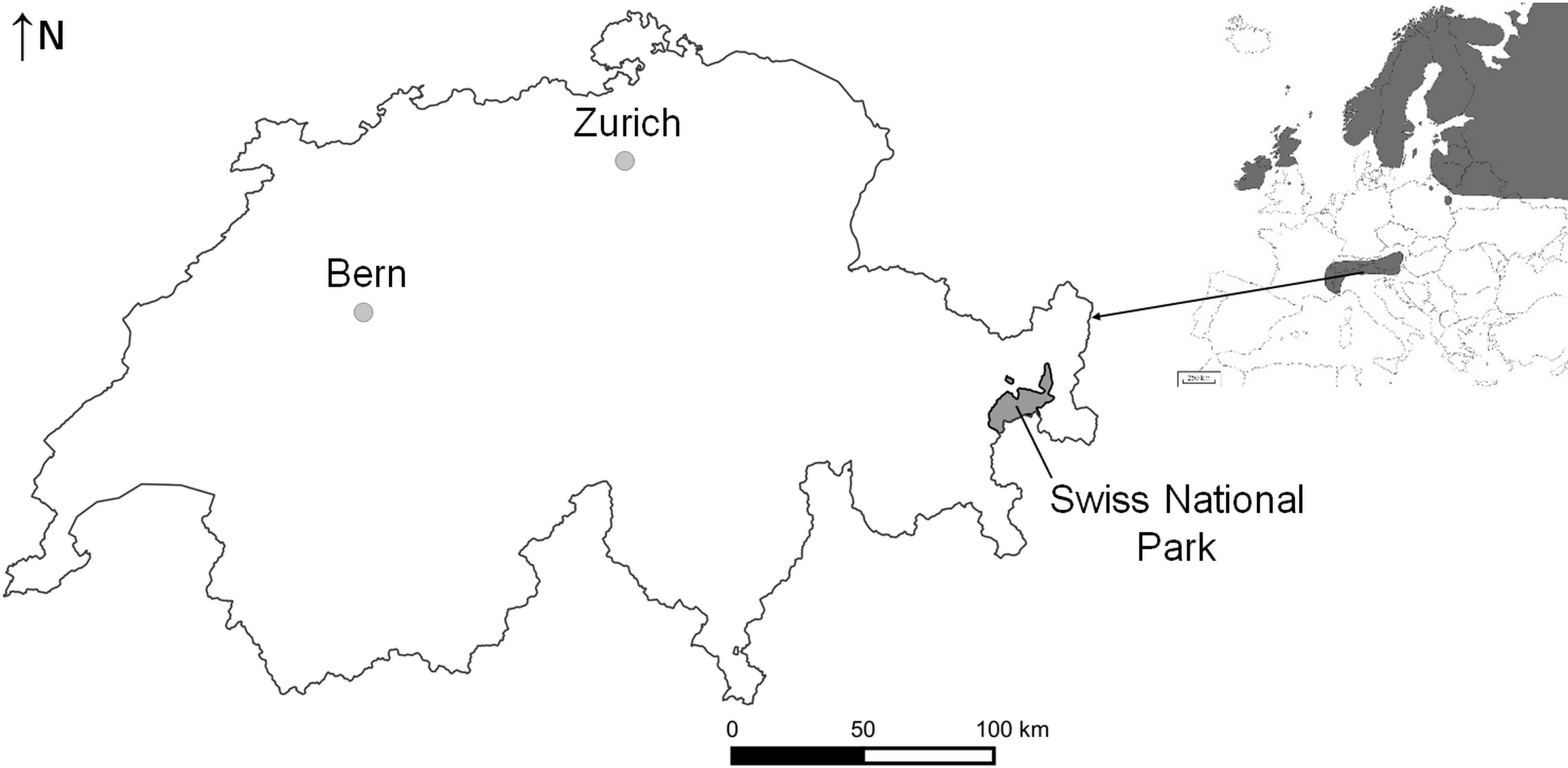
The distribution of mountain hares in Europe [28], and the location of the study area in the Swiss National Park (grey region) in Switzerland.

The study area is situated along the Ofenpass in the Swiss National Park in south-eastern Switzerland (46°39’N, 10°11’E; Fig 1). The estimated mountain hare density in spring 2014 was 3.4 mountain hares per 100 ha [16]. The Swiss National Park is designated by the International Union for the Conservation of Nature [27] as a Category 1a nature reserve (strict nature reserve/wilderness area) and is closed to the public in winter, usually until the second half of April. Thus, mountain hares can be studied under natural conditions without human disturbance during the mating period in spring and with minimal disturbance during the post-reproductive period in autumn. The research committee and the Department of Research and Geoinformation of the Swiss National Park gave permission to conduct the study on this site.

The 3.5 km^2^ study area ranges in elevation from 1693 to 2587 m a.s.l. The climate in the Swiss National Park is continental, with a mean January temperature of– 9°C and a mean July temperature of 11°C [29]. The monthly mean precipitation measured at 1970 m a.s.l. is 34 mm in January and 108 mm in July [29]. Habitat classification was based on the project HABITALP, which developed a habitat classification for protected areas in the Alps [30]. The study encompasses seven main habitat types: meadows (29%; with diverse grasses, including *Nardus stricta*, *Festuca* sp., *Poa* ssp., *Agrostis* ssp., *Luzula* ssp., and sedges), timber stands (24%), scree slopes (16%), storeyed stands (12%; mixed *Larix decidua*, *Pinus cembra*, *Pinus sylvestris*, *Pinus mugo* spp., *Picea abies*), sapling stands (6%; dominated by *Pinus mugo* spp.), pole timber (5%) and mature stands (5%). Other habitats cover 3% of the area.

### Sample collection

We collected fresh faeces during the mating period (end of March until first half of April) and the post-reproductive period (October) in 2014 and 2015. Samples were collected both systematically and opportunistically, as described in detail by Rehnus and Bollmann [16]. Systematic sampling was conducted on 91 plots that were pre-selected on a 200-m square grid. We removed all hare faeces from the trial plots and collected fresh faecal pellets from each plot three days later to ensure the stability of faecal metabolites [21]. For the opportunistic sampling, we collected fresh faeces when we moved from one systematic plot to the next one. Upon encountering fresh mountain hare tracks in the snow, we followed the tracks to the site of the next faeces deposition. Then, one faecal pellet was collected into a separate tube for DNA extraction to minimize contamination [31] and the rest was kept for GCM analysis. Thus, faecal pellets from the same defecation were used for both genetic and steroid analysis (but stored separately at -24°C until processed).

### Genetic method

The method used to genotype mountain hares is described in detail in Rehnus and Bollmann [16]. Briefly, we used ten microsatellite markers, which were successfully developed and applied under field conditions (faeces from mountain hares not older than 18 days in spring). DNA extraction from faeces was performed using the QIAamp^®^ Fast DNA Stool Mini Kit (QIAGEN, Hilden, Germany). Amplification was performed in three independent replicates in two multiplex PCRs at each of the ten microsatellites and at one sex-specific locus [16, 32]. For the identification of unique genotypes, we used CERVUS 3.0 [33].

### Analysis of faecal cortisol metabolites

Faecal GCM were measured using an 11-oxoaetiocholanolone enzyme immunoassay (EIA), which has proven suitable (based on results of a validation study including an ACTH challenge test) for evaluating adrenocortical activity in mountain hares [21]. Every sample was dried and thoroughly homogenized. Afterwards, a portion (0.15 g) was mixed with 5 ml methanol (80%), shaken (30 min) and centrifuged (2,500 g; 15 min) and an aliquot of the supernatant (after 1:10 dilution with assay buffer) analyzed in the 11-oxoaetiocholanolone EIA. All intra- and interassay coefficients of variation were below 12% and the sensitivity of the method was 2 ng/g faeces. Details of the extraction procedure and the EIA can be found elsewhere [2, 21, 34].

### Ethics statement

Faecal samples were collected without any handling of mountain hares.

### Statistics

All statistical tests were conducted using R 3.1.2 [35].

#### Influence of season and sex on GCM concentrations

We first investigated the effect of year and season on the GCM concentrations in the collected “anonymous” fresh faecal pellets to compare their results with findings that include genetic information at different levels (S1 Table). In a second step, we included first genetic information and analyzed the effect of sex on the GCM concentrations beside year and season. In a third step, we supplementary include genetic information of individuals in our analysis to avoid pseudo-replication. Thus, before the third analysis, we replaced all samples from one individual at the same sampling location and on the same day with their mean GCM concentration (S2 Table). Finally (further processing data from step three), we accounted for potential variations in resampling rate of unique individuals per season and year. Thereby we used means of the GCM concentrations of genotyped faeces collected from the same individuals in the same season and year.

One-way analysis of variance (ANOVA) were used to test for the influence of year (2014 and 2015), season (mating and post-reproductive periods), sex (male and female) and individual on GCM concentrations depending upon the level of integration of genetic information in the analysis. If we detected an influence of two variables, student’s t-tests were used to investigate the difference in GCM concentrations within the first variable (e.g. sex) across categories of the second variable (e.g. season). In order to get a normal distribution (Shapiro-Wilk normality test), the data were log-transformed before the first and second analysis. In the fourth analysis, we used Mann-Whitney U-tests instead of ANOVA to investigate the differences in GCM concentration among sex during spring and among season within females because data were not normal distributed after log-transformation.

#### Individual variability

First, we analysed patterns of resampling rate per individual to evaluate the risk of oversampling certain individuals in the collected fresh faecal samples. We used χ^2^ tests to evaluate the influence of year, season, sex and individual (as an indicator for individual behaviour) on the resamplings (yes/no) for individuals.

Secondly, we analyzed the variance in GCM concentrations within unique individuals per location within the mating and post-reproductive periods and within sexes. We used post-hoc tests to illustrate significant differences in GCM concentrations across unique individuals.

Finally, we compared our results to seasonal patterns in unique individuals. To do this, we used student's t-tests to investigate seasonal differences in GCM concentrations in the two individuals with the highest resampling rates (corrected to avoid pseudo-replicates of the same day and location) over both years: female (F2; resamplings = 13) and male (M5; resamplings = 7). Normal distribution was tested by Shapiro-Wilk normality test.

## Results

### Sample collection

In total, we collected 176 fresh, faecal samples from 38 individuals. Resampling rate for unique individuals varied strongly (Table 1).

**Table 1:** Origin (based on NIGS) of multiple faecal samples (N = 176) collected in the Swiss National Park during the mating and post-reproductive periods (2014 and 2015).

Parameter	Unit	All seasons	Spring	Autumn
Unique individuals	N	38	29	20
Females	N	17	14	9
Males	N	21	15	11
Individuals with resamplings	%	81.6	86.2	70.0
Average samples per individual	N	4.6	4.3	2.6
Maximal samples per individual	N	19	17	7

### Influence of season and sex on GCM concentrations

The effect of year, season, sex and individual varied dependent on the use of genetic information. In anonymous samples (N = 176), season had a significant influence on GCM concentration (F_1,175_ = 10.10, p = 0.002) while year did not (F_1,175_ = 2.97, p = 0.087). Higher concentrations (mean ± SE) were found in the mating period (104.4 ± 6.2 ng/g faeces) than in the post-reproductive period (69.4 ± 4.8 ng/g).

When genetic information on sex was included in the analysis of anonymous samples (N = 176) then the significant influence of season on GCM concentration remained (F_1,175_ = 10.21, p = 0.002), while sex (F_1,175_ = 2.88, p = 0.091) and year had no influence (F_1,175_ = 3.00, p = 0.085).

**Fig 2:**
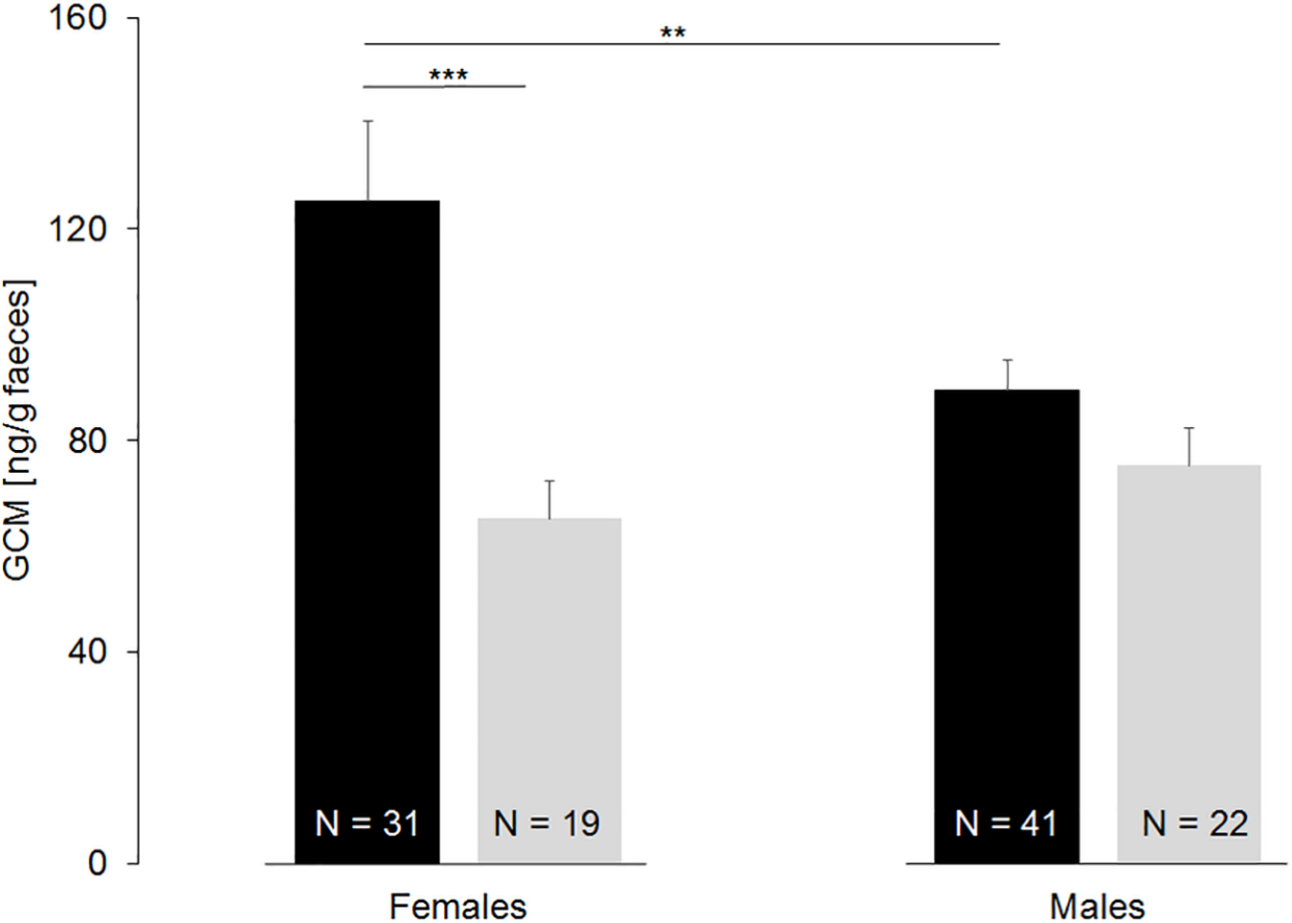
Concentrations (mean ± SE) of faecal glucocorticoid metabolites (GCM) in female and male mountain hares during the mating period (black) and the post-reproductive period (grey) for unique individuals per location (N = 113).

However, in the third analysis, where we added “individual” by genetic information (N = 113), we found that sex had a significant influence on GCM concentrations (F_1,112_ = 4.15, p = 0.045) in addition to season (F_1,112_ = 10.37, p = 0.002), while year (F_1,112_ = 1.17, p = 0.284) and individual had no influence (F_38,75_ = 1.18, p = 0.273). Females had higher GCM concentrations than males in the mating period, but not in the post-reproductive period (Fig 2). Within sexes, significantly higher GCM concentrations were found in females in the mating period as compared to the post-reproductive period (t = -3.693, p < 0.001). However, this seasonal pattern was not present in males (t = -1.405, p = 0.166; Fig 2).

To consider inter-individual variation in resampling rate of unique individuals per season and year our sample size was further reduced (N = 58). In this analysis influence of sex disappeared (F_1,57_ = 0.58, p = 0.456) while season had a significant influence on GCM concentration (F_1,57_ = 6.69, p = 0.018) and year (F_1,57_ = 0.06, p = 0.812) and individual had no influence (F_38,20_ = 0.72, p = 0.807). Nevertheless females had higher GCM concentrations than males in the mating period, but those differences were not significant (females: 124.5 ± 22.4 ng/g faeces; males: 85.7 ± 10.8 ng/g faeces; W = 209, p = 0.124). In the post-reproductive period the difference in GCM concentration among sex were not significant (t = -0.58, p = 0.566). Within sexes, significantly higher GCM concentrations were found in females in the mating period (124.5 ± 22.4 ng/g faeces) as compared to the post-reproductive period (60.2 ± 7.8 ng/g faeces, W = 32, p = 0.005). However, this seasonal pattern was not present in males (t = -0.72, p = 0.478).

### Individual variability

In total, we identified 38 unique individual mountain hares, 81.6% of which were resampled at least once (Table 1). When considering all samples (N = 176) resampling rates of genotyped individuals were neither influenced by season (χ^2^ = 1.1, df = 1, p = 0.306), nor by sex (χ^2^ = 0.0, df = 1, p = 1), nor by year (χ^2^ = 0.0, df = 1, p = 1), but depended on the individual animal (χ^2^ = 189, df = 37, p < 0.001).

**Fig 3:**
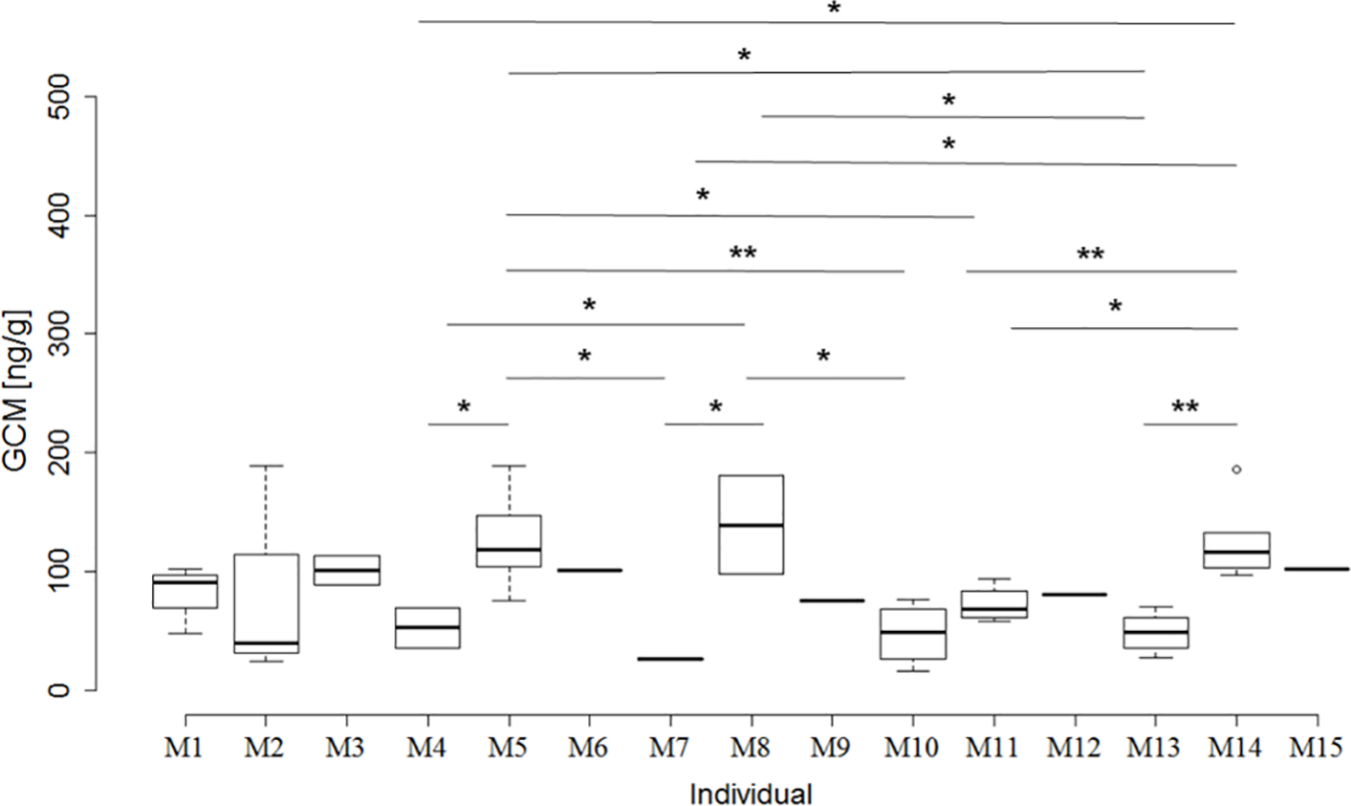
Individual variability in concentrations of faecal glucocorticoid metabolites (GCM) in male hare samples (in case of pseudo-replicates–more samples at the same location and on the same day–only the mean GCM is included) during the 2014 and 2015 mating periods (end of March until first half of April) in the Swiss National Park (N = 15; M1–M15). Post-hoc tests showed significant differences in GCM concentrations across individuals.

Within unique individuals per location within the mating and post-reproductive periods and within sexes (N = 113), we found that GCM concentrations were slightly dependent on the unique individual male during the mating period (F_1,14_ = 2.04, p = 0.056; Fig 3). The individual GCM levels differed up to 5-fold between individuals (median: 26.7–139.2 ng/g; Fig 3). We did not observe this influence in females during the mating period (females: F_1,13_ = 1.01, p = 0.485), nor in either of the sexes in the post-reproductive period (females: F_1,8_ = 2.31, p = 0.107; males: F_1,10_ = 1.75, p = 0.185).

Within sex for unique individuals per season (N = 58), we neither observed an influence of unique individuals during the mating period (females: F_1,13_ = 9.37, p = 0.101; males: F_1,14_ = 1.15, p = 0.476), nor in either of the sexes in the post-reproductive period (females: F_1,8_ = 0.96, p = 0.604; males: F_1,10_ = 42.20, p = 0.119).

To demonstrate variability of individual GCM concentrations in our comparison of the seasonal GCM pattern, we used the individual female F2 and the male M5 as examples; both had the highest resampling rate over both years per sex. Individual F2 confirmed the observation that females have higher GCM levels during the mating period as compared to the post-reproductive period (t = -3.48, p = 0.005; mean ± SE in the mating period: 168.7 ± 26.9 ng/g faeces and in the post-reproductive period: 73.4 ± 5.7 ng/g). However, the observation that males have similar GCM concentrations in the mating and the post-reproductive periods was not confirmed by individual M5. Instead M5 showed significantly higher GCM levels in the mating period as compared to the post-reproductive period (t = -3.44, p = 0.019; mean ± SE in the mating period: 127.2 ± 15.9 ng/g faeces and in the post-reproductive period: 64.4 ± 8.9 ng/g).

## Discussion

### Influence of season and sex on GCM concentrations

Our results showed how genetic data improve the interpretation of results of faecal glucocorticoid metabolite measurements in a free-living population of mountain hares. While the analysis of anonymous faeces showed that GCM concentrations were influenced by season only, genetic information indicates effects of sex and individual. However, the improvement of results by genetic information depends on the sample size from unique individuals at different location.

The effect of season may be explained by higher energetic costs of both sexes and all individuals during the mating season. However, sex and individual differences in this pattern cannot be estimated without knowledge of the composition of the sampled population which is necessary for an accurate interpretation of the results. When genetic information was added, we found higher GCM concentrations in females during the mating/gestation period as compared to the post-reproductive period. For females, the mating period is energetically costly due to the activation of catabolic processes associated with hypothalamic–pituitary–adrenal (HPA) axis activation, ovulation and pregnancy [36, 37]. Furthermore, we assume that GCM levels are higher when females reject potential mating partners. The rejection reaction is quite strong, as described by Hewson [38]: “It can be strong by strike and chase and she turned with lowered ears, struck with her forefeet, rarely making contact and chased the male for a few meters before settling down again to graze”. In addition, the physiological demands of placental function due to endocrine, paracrine and/or autocrine activity during pregnancy and fetal cardiovascular activity may result in higher GCM concentrations [39].

Interestingly, GCM concentrations in males are quite similar during both the mating and the post-reproductive periods. This is in contrast to studies that have shown higher GCM concentrations in males of other wildlife species during the mating season associated with reproductive and mating competition (chamois *Rupicapra rupicapra*: [6]; wolves *Canis lupus*: [40]. However, the mating season may be less costly for mountain hare males, because the species has a polygynandrous (promiscuous) mating system [28]. This may reduce the pressure to seek out and defend a single mate. Similarly, mountain hares and other Lagomorpha establish strong hierarchical systems prior to the mating season, which reduce mating competition across males [38, 41, 42]. For example, in European hare (*Lepus europaeus*), it was shown that the nearer a doe was to oestrus, the higher the rank of any consort was likely to be; no fighting or boxing between bucks was observed during the latter part of the breeding season [42].

### Individual variability

Despite the low number of samples in our study, the influence of unique individuals on GCM concentration within sex and season was partly close to being statistically signficant, which indicates potential pitfalls in the interpretation of anonymous samples by individual differences in GCM concentration and resampling rate.

We assume that the observed variance in GCM levels between individual males during the mating period is explained by differences in social status [6, 40, 43–45]. Higher-ranking males may have higher reproductive success than their lower-ranking counterparts, as has been shown for other wildlife species [46, 47]. The dominant hare often occupied the same area and chased away any subordinate counterparts [38]. However, individual differences in GCM excretion can also depend on factors such as behaviour, morphology, phenology, physiology and early (even prenatal) life experiences [10, 48–52].

Similarly, individual differences lead to significant differences in individual resampling rates. Thus, an oversampling (but also an undersampling) of individuals with different GCM levels can lead to misinterpretations of GCM results from “anonymous” faecal samples. Furthermore, a lack of knowledge about the identity of the individual animals hinders an understanding of individual life histories, and hence of the evolution of the population [10, 48, 49, 52, 53]. The variability of individual GCM concentrations is clearly demonstrated in our comparison of the seasonal GCM pattern of individual M5 versus that of the population. However, we also show high intra-individual variation in GCM, which can be explained by small sample size in our study, but also by other factors such as reproductive condition, predation or other acute stressors [1, 3, 23].

### Combining GCM measurements and NIGS

Anonymous faecal samples may cause overrepresentation of particular individuals thus introducing a source of error when estimating GCM levels within a population [9]. Over the last years NIGS has become more readily available and could be combined with GCM measurement to improve the interpretation of results of faecal glucocorticoid metabolite measurements in free-living populations. However, so far in free-ranging populations this was only applied to determine the species [19, 20] or sex [17] of the animal producing the faecal sample.

Individual-level variation (both at baseline and in response to stress) combined with sex and life history factors make combined population-level assessments of adrenocortical activity difficult. One way to overcome this challenge is to identify individuals from whom serial samples are collected. For species that are easier to observe directly, such as those that are diurnally active or who display sexual and individual variation in appearance (or are specially marked), GCM measurements can be made using a detailed collection protocol (e.g. documentation of sex or individual) for field sampling, as for example described in primates, elephants or chamois [54–56]. If only anonymous sampling can be performed, genetic analyses can help to specify serial samples from the same individuals. However, drawbacks are the higher costs and the reduced actual number of samples for running statistical analyses (e.g. detailed analysis of individual differences in GCM concentration or investigation of interactions among explaining variables).

## Conclusions

Our study showed that sex- and individual-dependent GCM levels can lead to misinterpretations of GCM values in “anonymous” faeces, especially during the mating period. For instance if the samples from subdominant males prevail in the mating period, seasonal GCM levels will be biased to GCM concentrations characteristic of post-reproductive period and sex and individual differences will be masked. To accurately evaluate the amount of stress experienced by free-living wild animals using faecal GCM measurements, we recommend genetic identification of sampled individuals. Such a combination allows one to remove outlier samples from individuals that are over- or under-sampled. Furthermore, combining NIGS and GCM can help identify sources of individual-level variation within a species that might be masked on the average population level, thus providing a key to understanding how the HPA-axis responds to ecological disturbances [57]. In stress-sensitive, elusive species without sexual dimorphism, this is possible using a combination of NIGS and GCM. Samples can be collected easily without any need to handle the animal and sampling is almost feedback free (not affected by collection itself). Furthermore, measured GCM levels can be attributed to unique individuals, avoiding pseudo-replication, but also allowing to specify serial samples from the same individuals.

## Supporting information

S1 Table Data used for the investigation of the effect of year and season on the GCM concentrations in the collected fresh faecal pellets (N = 176). (PDF)

S2 Table Data used for the investigation of the effect of year, season and individuals whereas all samples from one individual at the same sampling location and on the same day were recognized with their mean GCM concentration (N = 113). (PDF)

## References

[1] Dantzer B, Santicchia F, van Kesteren F, Palme R, Martinoli A, Wauters L. Measurement of faecal glucocorticoid metabolite levels in Eurasian red squirrels (*Sciurus vulgaris*): effects of captivity, sex, reproductive condition, and season. J Mammal. 2016; 97:1385–98. doi: 10.1093/jmammal/gyw095

[2] Möstl E, Maggs JL, Schrötter G, Besenfelder U, Palme R. Measurement of cortisol metabolites in faeces of ruminants. Vet Res Comm. 2002; 26:127–39. doi: 10.1023/A:1014095618125 . 11922482 10.1023/a:1014095618125

[3] Palme R. Measuring faecal steroids: guidelines for practical application. Ann NY Acad Sci. 2005; 1046:75–80. doi: 10.1196/annals.1343.007 . 16055844 10.1196/annals.1343.007

[4] Sheriff MJ, Dantzer B, Delehanty B, Palme R, Boonstra R. Measuring stress in wildlife: techniques for quantifying glucocorticoids. Oecologia. 2011; 166:869–87. doi: 10.1007/s00442-011-1943-y . 21344254 10.1007/s00442-011-1943-y

[5] Boonstra R. Reality as the leading cause of stress: rethinking the impact of chronic stress in nature. Funct Ecol. 2013; 27:11–23. doi: 10.1111/1365-2435.12008

[6] Corlatti L, Palme R, Lovari S. Physiological response to etho-ecological stressors in male Alpine chamois: timescale matters! Naturwissenschaften. 2014; 101:577–86. doi: 10.1007/s00114-014-1195-x . 24908399 10.1007/s00114-014-1195-x

[7] Goymann W. On the use of non-invasive hormone research in uncontrolled, natural environments: The problem with sex, diet, metabolic rate and the individual. Methods Ecol Evol. 2012; 3:757–65. doi: 10.1016/j.cbpa.2013.03.033 PMID: 23562802.

[8] Hadinger U, Haymerle A, Knauer F, Schwarzenberger F, Walzer C. Faecal cortisol metabolites to assess stress in wildlife: evaluation of a field method in free-ranging chamois. Methods Ecol Evol. 2015; 6:1349–57. doi: 10.1111/2041-210x.12422

[9] Huber S, Palme R, Arnold W. Effects of season, sex, and sample collection on concentrations of fecal cortisol metabolites in red deer (*Cervus elaphus*). Gen Comp Endocr. 2003; 130:48–54. doi: 10.1016/S0016-6480(02)00535-X . 12535624 10.1016/s0016-6480(02)00535-x

[10] Sapolsky RM. Adrenocortical function, social rank, and personality among wild baboons. Biol Psychiatry. 1990; 28:862–78. doi: 10.1016/0006-3223(90)90568-m . 2268690 10.1016/0006-3223(90)90568-m

[11] Taberlet P, Luikart G. Non-invasive genetic sampling and individual identification. Biol J Linn Soc. 1999; 68:41–55. doi: 10.1111/j.1095-8312.1999.tb01157.x

[12] Goossens B, Bruford MW. Non-invasive genetic analysis in conservation In: Bertorelle G, Bruford M.W., Hauffe H.C., Rizzoli A., Vernisi C., editor. Population Genetics for Animal Conservation. Cambridge: Cambridge University Press; 2009 p. 167–201.

[13] Schwartz MK, Luikart G, Waples RS. Genetic monitoring as a promising tool for conservation and management. Trends Ecol Evol. 2007; 22:25–33. doi: 10.1016/j.tree.2006.08.009 . 16962204 10.1016/j.tree.2006.08.009

[14] Waits LP, Paetkau D. Noninvasive genetic sampling tools for wildlife biologists: A review of applications and recommendations for accurate data collection. J Wildl Manage. 2005; 69:1419–33. doi: 10.2193/0022-541x

[15] Jacob G, Debrunner R, Gugerli F, Schmid B, Bollmann K. Field surveys of capercaillie (*Tetrao urogallus*) in the Swiss Alps underestimated local abundance of the species as revealed by genetic analyses of non-invasive samples. Conserv Genet. 2010; 11:33–44. doi: 10.1007/s10592-008-9794-8

[16] Rehnus M, Bollmann K. Non-invasive genetic population monitoring of mountain hares (*Lepus timidus*) in the Alps: systematic or opportunistic sampling? Eur J Wildl Res. 2016; 62:737–47. doi: 10.1007/s10344-016-1053-6

[17] Joly K, Wasser SK, Booth R. Non-invasive assessment of the interrelationships of diet, pregnancy rate, group composition, and physiological and nutritional stress of Barren-Ground Caribou in late winter. Plos One. 2015; 10 doi: 10.1371/journal.pone.0127586 . 26061003 10.1371/journal.pone.0127586 PMC4464525

[18] Mesa-Cruz JB, Brown JL, Waits LP, Kelly MJ. Non-invasive genetic sampling reveals diet shifts, but little difference in endoparasite richness and faecal glucocorticoids, in Belizean felids inside and outside protected areas. J Trop Ecol. 2016; 32:226–39. doi: 10.1017/s0266467416000213

[19] Wasser SK, Davenport B, Ramage ER, Hunt KE, Parker M, Clarke C, et al Scat detection dogs in wildlife research and management: application to grizzly and black bears in the Yellowhead Ecosystem, Alberta, Canada. Can J Zool. 2004; 82:475–92. doi: 10.1139/z04-020

[20] Wasser SK, Keim JL, Taper ML, Lele SR. The influences of wolf predation, habitat loss, and human activity on caribou and moose in the Alberta oil sands. Front Ecol Evol. 2011; 9:546–51. doi: 10.1890/100071

[21] Rehnus M, Hackländer K, Palme R. A non-invasive method for measuring glucocorticoid metabolites (GCM) in Mountain hares (*Lepus timidus*). Eur J Wildl Res. 2009; 55:615–20. doi: 10.1007/s10344-009-0297-9

[22] Rehnus M, Palme R, Filli F, Hackländer K. Seasonal glucocorticoid secretion in mountain hares (*Lepus timidus*). Mammalia. 2010; 74:347–50. doi: 10.1515/mamm.2010.032

[23] Rehnus M, Wehrle M, Palme R. Mountain hares *Lepus timidus* and tourism: stress events and reactions. J Appl Ecol. 2014; 51:6–12. doi: 10.1111/1365-2664.12174

[24] Rehnus M. Der Schneehase in den Alpen. Ein Überlebenskünstler mit ungewisser Zukunft. Bern Stuttgart Wien: Haupt; 2013.

[25] Thulin CG. The distribution of mountain hares *Lepus timidus* in Europe: a challenge from brown hares *L*. *europaeus*? Mammal Rev. 2003; 33:29–42.

[26] Bisi F, Nodari M, Dos Santos Oliviera NM, Masseroni E, Preatoni DG, Wauters LA, et al Space use patterns of mountain hare (*Lepus timidus*) on the Alps. Eur J Wildl Res. 2011; 57:305–12.

[27] IUCN. Protected Areas Category Ia Gland: IUCN; 2016 [19 th October 2016]. Available from: https://www.iucn.org/about/work/programmes/gpap_home/gpap_quality/gpap_pacategories/gpap_cat1a/.

[28] Thulin C-G, Flux JEC. *Lepus timidus* Linnaeus, 1758 –Schneehase In: Krapp F, editor. Handbuch der Säugetiere Europas Band, 3/II: Hasenartige Lagomorpha. Wiebelsheim: Aula Publisher; 2003 p. 155–85.

[29] Haller H, Eisenhut A, Haller R. Atlas des Schweizerischen Nationalparks. Die ersten 100 Jahre. Bern: Haupt; 2013.

[30] Lotz A, editor. Alpine Habitat Diversity—HABITALP—Project Report 2002–2006. Berchtesgaden: Nationalpark Berchtesgaden; 2006.

[31] Sloane MA, Sunnucks P, Alpers D, Beheregaray LB, Taylor AC. Highly reliable genetic identification of individual northern hairy-nosed wombats from single remotely collected hairs: A feasible censusing method. Mol Ecol. 2000; 9:1233–40. doi: 10.1046/j.1365-294x.2000.00993.x . 10972763 10.1046/j.1365-294x.2000.00993.x

[32] Wallner B, Huber S, Achmann R. Non-invasive PCR sexing of rabbits (*Oryctolagus cuniculus*) and hares (*Lepus europaeus*). Mamm Biol. 2001; 66:190–2.

[33] Kalinowski ST, Taper M, Marshall TC. Revising how the computer program cervus accommodates genotyping error increases success in paternity assignment. Mol Ecol. 2007; 16:1099–106. doi: 10.1111/j.1365-294X.2007.03089.x . 17305863 10.1111/j.1365-294X.2007.03089.x

[34] Palme R, Touma C, Arias N, Dominchin MF, Lepschy M. Steroid extraction: Get the best out of faecal samples. Wien Tierarztl Monatsschr. 2013; 100:238–46.

[35] R Development Core Team. R: A language and environment for statistical computing Vienna: R Foundation for Statistical Computing; 2016.

[36] Cavigelli SA, Dubovick T, Levash W, Jolly A, Pitts A. Female dominance status and fecal corticoids in a cooperative breeder with low reproductive skew: ring-tailed lemurs (*Lemur catta*). Horm Behav. 2003; 43:166–79. doi: 10.1016/s0018-506x(02)00031-4 . 12614647 10.1016/s0018-506x(02)00031-4

[37] Saltzman W, Schultz-Darken NJ, Wegner FH, Wittwer DJ, Abbott DH. Suppression of cortisol levels in subordinate female marmosets: Reproductive and social contributions. Horm Behav. 1998; 33:58–74. doi: 10.1006/hbeh.1998.1436 9571014 10.1006/hbeh.1998.1436

[38] Hewson R. Behaviour, population changes and dispersal of mountain hares (*Lepus timidus*) in Scottland. J Zool Lond. 1990; 220:287–309. doi: 10.1111/j.1469-7998.1990.tb04309.x

[39] Sun K, Yang K, Challis JRG. Glucocorticoid actions and metabolism in pregnancy: Implications for placental function and fetal cardiovascular activity. Placenta. 1998; 19:353–60. doi: 10.1016/s0143-4004(98)90074-1 . 9699955 10.1016/s0143-4004(98)90074-1

[40] Sands J, Creel S. Social dominance, aggression and faecal glucocorticoid levels in a wild population of wolves, *Canis lupus*. Anim Behav. 2004; 67:387–96. doi: 10.1016/j.anbehav.2003.03.019

[41] Graf RP. Social-organization of snowshoe hares. Can J Zool. 1985; 63:468–74. doi: 10.1139/z85-066

[42] Holley AJF. A hierarchy of hares: dominance status and access to oestrus does. Mammal Rev. 1986; 16:181–6. doi: 10.1111/j.1365-2907.1986.tb00040.x

[43] Cavigelli SA, Caruso MJ. Sex, social status and physiological stress in primates: the importance of social and glucocorticoid dynamics. Philos Trans R Soc B. 2015; 370 doi: 10.1098/rstb.2014.0103 . 25870390 10.1098/rstb.2014.0103 PMC4410370

[44] Goymann W, East ML, Wachter B, Honer OP, Mostl E, Van't Hof TJ, et al Social, state-dependent and environmental modulation of faecal corticosteroid levels in free-ranging female spotted hyenas. Proc R Soc Lond B. 2001; 268:2453–9. doi: 10.1098/rspb.2001.1828 . 11747563 10.1098/rspb.2001.1828 PMC1088899

[45] Mooring MS, Patton ML, Lance VA, Hall BM, Schaad EW, Fetter GA, et al Glucocorticoids of bison bulls in relation to social status. Horm Behav. 2006; 49:369–75. doi: 10.1016/j.yhbeh.2005.08.008 . 16257404 10.1016/j.yhbeh.2005.08.008

[46] Alonso JC, Magana M, Palacin C, Martin CA. Correlates of male mating success in great bustard leks: the effects of age, weight, and display effort. Behav Ecol Sociobiol. 2010; 64:1589–600. doi: 10.1007/s00265-010-0972-6

[47] Clutton-Brock TH, Huchard E. Social competition and selection in males and females. Philos Trans R Soc B. 2013; 368 doi: 10.1098/rstb.2013.0074 . 24167304 10.1098/rstb.2013.0074 PMC3826203

[48] Gosling SD. From mice to men: What can we learn about personality from animal research? Psychol Bull. 2001; 127:45–86. doi: 10.1037//0033-2909.127.1.45 . 11271756 10.1037/0033-2909.127.1.45

[49] Gross MR. Alternative reproductive strategies and tactics: Diversity within sexes. Trends Ecol Evol. 1996; 11:92–8. doi: 10.1016/0169-5347(96)81050-0 . 21237769 10.1016/0169-5347(96)81050-0

[50] Noer CL, Needham EK, Wiese AS, Balsby TJS, Dabelsteen T. Personality matters: Consistency of inter-individual variation in shyness-boldness across non-breeding and pre-breeding season despite a fall in general shyness levels in farmed American mink (*Neovison vison*). Appl Anim Behav Sci. 2016; 181:191–9. doi: 10.1016/j.applanim.2016.05.003 PMID: 26087277.

[51] Schöpper H, Palme R, Ruf T, Huber S. Effects of prenatal stress on hypothalamic-pituitary-adrenal (HPA) axis function over two generations of guinea pigs (*Cavia aperea f*. *porcellus*). Gen Comp Endocr. 2012; 176:18–27. doi: 10.1016/j.ygcen.2011.12.010 . 22202601 10.1016/j.ygcen.2011.12.010

[52] Smith BR, Blumstein DT. Fitness consequences of personality: a meta-analysis. Behav Ecol. 2008; 19:448–55. doi: 10.1093/beheco/arm144

[53] Forsman A, Wennersten L. Inter-individual variation promotes ecological success of populations and species: evidence from experimental and comparative studies. Ecography. 2016; 39:630–48. doi: 10.1111/ecog.01357

[54] Corlatti L, Béthaz S, von Hardenberg A, Bassano B, Palme R, Lovari S. Hormones, parasites and male reproductive tactics in Alpine chamois: identifying the mechanisms of life history trade-offs. Anim Behav. 2012; 84:1061–70. doi: 10.1016/j.anbehav.2012.08.005

[55] Ganswindt A, Münscher S, Henley M, Henley S, Heistermann M, Palme R, et al Endocrine correlates of musth and the impact of ecological and social factors in free-ranging African elephants (*Loxodonta africana*). Horm Behav. 2010; 57:506–14. doi: 10.1016/j.yhbeh.2010.02.009 . 20188104 10.1016/j.yhbeh.2010.02.009

[56] Wasserman MD, Chapman CA, Milton K, Gogarten JF, Wittwer DJ, Ziegler TE. Estrogenic plant consumption predicts red colobus monkey (*Procolobus rufomitratus*) hormonal state and behavior. Horm Behav. 2012; 62:553–62. doi: 10.1016/j.yhbeh.2012.09.005 . 23010620 10.1016/j.yhbeh.2012.09.005 PMC3513326

[57] Arlettaz R, Nussle S, Baltic M, Vogel P, Palme R, Jenni-Eiermann S, et al Disturbance of wildlife by outdoor winter recreation: allostatic stress response and altered activity-energy budgets. Ecol Appl. 2015; 25:1197–212. doi: 10.1890/14-1141.1 26485949 10.1890/14-1141.1

