# Supplementary material for: How genetic data improve the interpretation of results of faecal glucocorticoid metabolite measurements in a free-living population
Source: PLoS One. 2017 Aug 23;12(8):e0183718. doi: 10.1371/journal.pone.0183718 (PMC5568376; doi:10.1371/journal.pone.0183718)
Supplement: S1 Table — (PDF) [file pone.0183718.s001.pdf]

individual	sex	GCM	season	year
F1	female	102.399	spring	2014
F2	female	197.274	spring	2014
M1	male	47.8665	spring	2014
F3	female	267.2835	spring	2014
F3	female	472.7745	spring	2014
M2	male	180.675	spring	2014
M3	male	84.48	spring	2014
M3	male	66.8415	spring	2014
F4	female	96.9705	spring	2014
F4	female	119.625	spring	2014
F4	female	52.668	spring	2014
F5	female	72.996	spring	2014
F5	female	66.2805	spring	2014
F5	female	90.42	spring	2014
F3	female	92.0535	spring	2014
F3	female	128.403	spring	2014
M4	male	60.885	spring	2014
M5	male	99.165	spring	2014
M5	male	91.6245	spring	2014
M5	male	86.3445	spring	2014
M5	male	79.6125	spring	2014
M5	male	114.081	spring	2014
F6	female	3.828	spring	2014
M6	male	78.507	spring	2014
M6	male	62.0895	spring	2014
M6	male	100.815	spring	2014
M7	male	49.731	spring	2014
M7	male	41.1675	spring	2014
F7	female	113.8995	spring	2014
F7	female	174.9165	spring	2014
F7	female	137.709	spring	2014
F7	female	122.4795	spring	2014
F7	female	158.565	spring	2014
M8	male	139.5735	spring	2014
M8	male	92.664	spring	2014
M1	male	91.3605	spring	2014
F8	female	89.1495	spring	2014
F8	female	115.797	spring	2014
F8	female	71.973	spring	2014
F8	female	182.985	spring	2014
F9	female	68.904	spring	2014
F9	female	101.31	spring	2014
F9	female	132.363	spring	2014
F2	female	48.0645	spring	2014
F2	female	93.9015	spring	2014
M9	male	170.016	spring	2014
M9	male	34.551	spring	2014
M7	male	20.5425	spring	2014
M7	male	29.5845	spring	2014

M7	male	31.185	spring	2014
F10	female	181.1205	spring	2014
F10	female	20.5425	spring	2014
M7	male	61.248	spring	2014
M7	male	79.4145	spring	2014
M5	male	73.821	spring	2014
M4	male	36.9765	spring	2014
M10	male	39.699	spring	2014
F2	female	221.43	spring	2014
M5	male	56.892	spring	2014
M5	male	56.166	spring	2014
M5	male	61.644	spring	2014
M7	male	70.7685	spring	2014
M7	male	33.9075	spring	2014
M5	male	51.0345	spring	2014
M5	male	51.744	spring	2014
M5	male	99.033	spring	2014
M5	male	64.449	spring	2014
M5	male	52.635	spring	2014
F11	female	39.0225	spring	2014
F11	female	173.6295	spring	2014
F2	female	119.9055	spring	2014
F2	female	95.1225	spring	2014
F6	female	73.227	spring	2014
F6	female	68.1945	spring	2014
F12	female	65.439	spring	2014
M11	male	112.0185	autumn	2014
M12	male	63.228	autumn	2014
M12	male	63.228	autumn	2014
F13	female	20.856	autumn	2014
F10	female	31.086	autumn	2014
M1	male	61.1655	autumn	2014
F14	female	54.6645	autumn	2014
F14	female	89.6775	autumn	2014
F3	female	94.512	autumn	2014
M10	male	24.321	spring	2015
M13	male	89.1825	spring	2015
M14	male	69.9765	spring	2015
F6	female	43.4445	spring	2015
F6	female	42.7185	spring	2015
M15	male	171.7485	spring	2015
M15	male	140.9265	spring	2015
F2	female	272.382	spring	2015
F2	female	342.408	spring	2015
M16	male	100.815	spring	2015
F2	female	221.661	spring	2015
F2	female	185.4435	spring	2015
F2	female	226.314	spring	2015
F2	female	235.092	spring	2015
F2	female	258.225	spring	2015

M15	male	188.6775	spring	2015
M15	male	113.751	spring	2015
M15	male	124.3605	spring	2015
M8	male	186.0375	spring	2015
M8	male	132.891	spring	2015
M15	male	115.8465	spring	2015
M15	male	128.8155	spring	2015
M15	male	148.335	spring	2015
M15	male	136.0755	spring	2015
M4	male	16.5	spring	2015
M2	male	97.6635	spring	2015
F2	female	86.2125	spring	2015
M10	male	188.991	spring	2015
M1	male	102.5475	spring	2015
M8	male	99.165	spring	2015
M8	male	107.3325	spring	2015
M15	male	75.273	spring	2015
M8	male	117.909	spring	2015
M8	male	77.22	spring	2015
M15	male	92.6145	spring	2015
F15	female	76.395	spring	2015
F15	female	47.7675	spring	2015
F6	female	68.244	spring	2015
F16	female	134.739	spring	2015
F16	female	42.966	spring	2015
F6	female	31.746	spring	2015
F6	female	35.706	spring	2015
F6	female	89.925	spring	2015
M17	male	26.664	spring	2015
F2	female	81.807	spring	2015
F2	female	116.6055	spring	2015
F2	female	134.1285	spring	2015
M13	male	113.0745	spring	2015
M14	male	35.6895	spring	2015
F14	female	170.016	autumn	2015
F14	female	23.628	autumn	2015
M4	male	76.494	spring	2015
M18	male	72.798	autumn	2015
M18	male	80.883	autumn	2015
F2	female	67.6995	autumn	2015
F3	female	63.0135	autumn	2015
F7	female	43.23	autumn	2015
F7	female	42.9825	autumn	2015
F17	female	68.0955	autumn	2015
F2	female	79.068	autumn	2015
M19	male	49.7805	autumn	2015
M20	male	80.916	autumn	2015
M20	male	86.1135	autumn	2015
F6	female	86.8065	autumn	2015
F11	female	35.013	autumn	2015

F3	female	67.3365	autumn	2015
F3	female	53.658	autumn	2015
F17	female	122.628	autumn	2015
M21	male	75.7515	autumn	2015
M21	male	99.3465	autumn	2015
M21	male	166.98	autumn	2015
M21	male	128.5515	autumn	2015
M21	male	95.7165	autumn	2015
M8	male	55.5225	autumn	2015
M8	male	63.261	autumn	2015
M8	male	83.4405	autumn	2015
M1	male	55.539	autumn	2015
M1	male	99.825	autumn	2015
M14	male	32.6535	autumn	2015
M18	male	46.332	autumn	2015
M18	male	18.6285	autumn	2015
M18	male	36.465	autumn	2015
M1	male	26.565	autumn	2015
F3	female	48.279	autumn	2015
F3	female	45.705	autumn	2015
F3	female	77.121	autumn	2015
F6	female	22.308	autumn	2015
F6	female	97.1025	autumn	2015
M13	male	39.468	autumn	2015
M13	male	97.1025	autumn	2015
M4	male	26.499	autumn	2015
M4	male	114.675	autumn	2015
