# Supplementary material for: How genetic data improve the interpretation of results of faecal glucocorticoid metabolite measurements in a free-living population
Source: PLoS One. 2017 Aug 23;12(8):e0183718. doi: 10.1371/journal.pone.0183718 (PMC5568376; doi:10.1371/journal.pone.0183718)
Supplement: S2 Table — (PDF) [file pone.0183718.s002.pdf]

sample_ID	individual	sex	GCM	season	year
1	F1	female	102.399	spring	2014
2	F2	female	197.274	spring	2014
3	M1	male	47.8665	spring	2014
4	F3	female	370.029	spring	2014
5	M2	male	180.675	spring	2014
6	M3	male	75.66075	spring	2014
7	F4	female	89.7545	spring	2014
8	F5	female	76.5655	spring	2014
9	F3	female	110.22825	spring	2014
10	M4	male	60.885	spring	2014
11	M5	male	94.1655	spring	2014
12	F6	female	3.828	spring	2014
13	M6	male	80.4705	spring	2014
14	M7	male	45.44925	spring	2014
15	F7	female	137.251125	spring	2014
16	F7	female	158.565	spring	2014
17	M8	male	116.11875	spring	2014
18	M1	male	91.3605	spring	2014
19	F8	female	114.976125	spring	2014
20	F9	female	100.859	spring	2014
21	F2	female	70.983	spring	2014
22	M9	male	102.2835	spring	2014
23	M7	male	27.104	spring	2014
24	F10	female	100.8315	spring	2014
25	M7	male	70.33125	spring	2014
26	M5	male	73.821	spring	2014
27	M4	male	36.9765	spring	2014
28	M10	male	39.699	spring	2014
29	F2	female	221.43	spring	2014
30	M5	male	58.234	spring	2014
31	M7	male	52.338	spring	2014
32	M5	male	63.7791	spring	2014
33	F11	female	106.326	spring	2014
34	F2	female	107.514	spring	2014
35	F6	female	70.71075	spring	2014
36	F12	female	65.439	spring	2014
37	M11	male	112.0185	autumn	2014
38	M12	male	63.228	autumn	2014
39	F13	female	20.856	autumn	2014
40	F10	female	31.086	autumn	2014
41	M1	male	61.1655	autumn	2014
42	F14	female	54.6645	autumn	2014
43	F14	female	89.6775	autumn	2014
44	F3	female	94.512	autumn	2014
45	M10	male	24.321	spring	2015
46	M13	male	89.1825	spring	2015
47	M14	male	69.9765	spring	2015
48	F6	female	43.0815	spring	2015
49	M15	male	156.3375	spring	2015

50	F2	female	272.382	spring	2015
51	F2	female	342.408	spring	2015
52	M16	male	100.815	spring	2015
53	F2	female	225.3471	spring	2015
54	M15	male	188.6775	spring	2015
55	M15	male	119.05575	spring	2015
56	M8	male	186.0375	spring	2015
57	M8	male	132.891	spring	2015
58	M15	male	115.8465	spring	2015
59	M15	male	137.742	spring	2015
60	M4	male	16.5	spring	2015
61	M2	male	97.6635	spring	2015
62	F2	female	86.2125	spring	2015
63	M10	male	188.991	spring	2015
64	M1	male	102.5475	spring	2015
65	M8	male	103.24875	spring	2015
66	M15	male	75.273	spring	2015
67	M8	male	97.5645	spring	2015
68	M15	male	92.6145	spring	2015
69	F15	female	76.395	spring	2015
70	F15	female	47.7675	spring	2015
71	F6	female	68.244	spring	2015
72	F16	female	134.739	spring	2015
73	F16	female	50.08575	spring	2015
74	M17	male	26.664	spring	2015
75	F2	female	81.807	spring	2015
76	F2	female	116.6055	spring	2015
77	F2	female	134.1285	spring	2015
78	M13	male	113.0745	spring	2015
79	M14	male	35.6895	spring	2015
80	F14	female	96.822	autumn	2015
81	M4	male	76.494	spring	2015
82	M18	male	76.8405	autumn	2015
83	F2	female	67.6995	autumn	2015
84	F3	female	63.0135	autumn	2015
85	F7	female	43.23	autumn	2015
86	F7	female	42.9825	autumn	2015
87	F17	female	68.0955	autumn	2015
88	F2	female	79.068	autumn	2015
89	M19	male	49.7805	autumn	2015
90	M20	male	80.916	autumn	2015
91	M20	male	86.1135	autumn	2015
92	F6	female	86.8065	autumn	2015
93	F11	female	35.013	autumn	2015
94	F3	female	60.49725	autumn	2015
95	F17	female	122.628	autumn	2015
96	M21	male	75.7515	autumn	2015
97	M21	male	99.3465	autumn	2015
98	M21	male	166.98	autumn	2015
99	M21	male	128.5515	autumn	2015

100	M21	male	95.7165	autumn	2015
101	M8	male	55.5225	autumn	2015
102	M8	male	73.35075	autumn	2015
103	M1	male	55.539	autumn	2015
104	M1	male	99.825	autumn	2015
105	M14	male	32.6535	autumn	2015
106	M18	male	39.49275	autumn	2015
107	M18	male	36.465	autumn	2015
108	M1	male	26.565	autumn	2015
109	F3	female	46.992	autumn	2015
110	F3	female	77.121	autumn	2015
111	F6	female	59.70525	autumn	2015
112	M13	male	68.28525	autumn	2015
113	M4	male	70.587	autumn	2015
